# Outcome Analysis of Distal End Radius Fractures Managed With Antegrade Intramedullary K-wire Fixation

**DOI:** 10.7759/cureus.30512

**Published:** 2022-10-20

**Authors:** Abhiram Awasthi, Shivshankar Jadhav, Shounak Taywade, Ankur Salwan, Khizar Khan

**Affiliations:** 1 Department of Orthopaedic Surgery, Jawaharlal Nehru Medical College, Datta Meghe Institute of Medical Sciences, Wardha, IND

**Keywords:** intramedullary k-wire, functional outcome, fracture radius, pinning technique, k-wire

## Abstract

Background

The most frequent upper limb fractures are distal end radius fractures, accounting for around 17% of all fractures in clinical practice. Falling on an outstretched hand is the most common mechanism of injury, and it can also occur in high-energy trauma in young individuals. A minimally invasive technique of percutaneous pinning was introduced to sustain the fracture’s reduction after manipulation and avoid the re-displacement of fractured fragments. Antegrade intramedullary K-wire fixation is a cost-efficient procedure that can be done in rural settings.

Methodology

A total of 30 patients with fractures of the distal end radius managed with antegrade intramedullary K-wire fixation were included in the study. Operated patients were followed up at one month, three months, and six months for functional assessment. An X-ray was taken on every follow-up to assess the union and implant positioning.

Results

In our study, the mean age was 45.6 years. Out of the 30 patients, 12 were males and 18 were females. All 30 patients at the final follow-up showed good functional improvement, with statistically significant improvements in palmar flexion, adduction and abduction, and pain scale scores.

Conclusions

Antegrade K-wire fixation is an effective technique for fractures of the distal end radius that can be performed in rural settings with effective results.

## Introduction

About 200 years ago, Abraham Colle was the first to describe distal end radius fractures and wrist fractures in the carpel extremity of radial fractures in 1814. However, even now the percentage of unsatisfactory results in the treatment of distal end radius fractures appears to be higher with increased injury-associated morbidities [1-3]. Colle first described that the wrist finally achieves freedom even without intervention in all movements and can be completely free of pain [4]. However, in most of these fractures, the distal radioulnar joint (DRUJ) or the radiocarpal joint is involved which can be initially reduced with manipulation; however, due to the instability, it collapses with time in simple cast immobilization. The most frequent fractures of the upper limb are distal end radius fractures, which account for around 17% of all fractures in clinical practice [5]. Falling on an outstretched hand position is the most common method of injury, but it is also common in high-energy traumas. Even though various treatment options are available, there is no consensus on the best way to treat these injuries. Furthermore, the radiological characteristics of reduction do not always correspond to the functional outcome.

The following are the most critical radiological factors that influence the outcome: ulnar variance, radial height, carpal alignment, palmar tilt, and articular congruity. Hence, getting these radiological parameters right is among the only factors in determining the eventual outcome of these fractures. Fractures of the distal end radius can be managed by different treatment modalities. These range from closed reduction and casting or K-wiring to open reduction and internal fixation (ORIF) with plate osteosynthesis to external fixators. ORIF with plate osteosynthesis is the gold standard technique for fractures of the distal end radius because it can restore the intraarticular alignment and volar tilt while providing rigid and secure fixation. It reduces the number of follow-ups and loss of working hours. It lowers the incidence of posttraumatic osteoarthritis [6,7]. Despite its advantages, volar plating has been associated with irritation to the flexor tendons or tendon rupture, tenosynovitis, which mandates hardware removal, as well as a complex regional pain syndrome (CRPS).

For stable intra-articular fractures and extra-articular fractures of the distal end radius, Sato et al. [8] proposed a reduction and antegrade K-wire fixing technique for extra-articular distal end radius fractures. Antegrade intramedullary K-wire fixation is a cost-efficient procedure that can be done in rural settings. The purpose of this research is to assess the functional and radiological outcomes of antegrade K-wire fixation for distal end radius fractures.

## Materials and methods

Between September 2019 and December 2021, patients with extra-articular distal end radius fractures were included in the study and managed with closed reduction and percutaneous antegrade intramedullary K-wire fixation. A total of 30 patients who met the inclusion criteria for the study were treated with this modality during the span of one year. Institutional Ethics Committee approval was taken from the Datta Meghe Institute of Medical Sciences Ethics Committee (reference number: DMIMS(DU)/IEC/Sept-2019/8352).

The patients were followed up on the immediate postoperative day and then after one month, three months, and on the final follow-up after six months. During the follow-up, X-rays were taken and evaluated based on the radial height and inclination. The pain and disability of the affected upper limb were evaluated based on the Visual Analog Scale (VAS) and the Disabilities of Arm Shoulder and Hand (DASH) scoring, respectively. Movements of the wrist were evaluated, and degrees of flexion, extension, abduction, adduction, pronation, and supination were noted.

Preoperative preparation of patients

Assessment of distal end radius fractures was done concerning skin condition (closed/open fracture), peripheral circulation, neurologic examination, especially median nerve, flexor and extensor tendon function, distal radioulnar joint stability, compartment syndrome, and associated injuries. Radiographs of the injured wrist were taken in the anteroposterior (AP) and lateral views.

The following radiographic parameters were noted: radial inclination in the AP view, radial length in the posteroanterior (PA) view, articular step-off/displacement, and then fractures were classified according to Frykman’s classification.

Operative procedure

The patient’s forearm was kept parallel to the ground on a radiolucent side table. Following standard sterilization and draping, a 3-cm longitudinal skin incision was made on the dorsomedial aspect of the mid-radius, about 7-10 cm proximal to the radial styloid. The incision was made just proximal to the abductor pollicis longus muscle between the extensor carpi radialis brevis and the extensor digitorum communis muscle. The cortex of the radius dorsal to the insertion of the pronator muscle was then exposed. In large-sized bones, one slanting hole was made with a 3.5-mm drill bit, assuming that the diameter of the drill hole was less than half the breadth of the bone at the level of drilling. To minimize the stress riser effect in patients with small bones, two drill holes at two separate levels were drilled using a 2-mm drill bit. The drill bit was initially guided perpendicular to the bone, then obliquely at an angle of 45-60°, taking care not to penetrate the distant cortex. Two 1.5-mm K-wires were manually inserted into the medullary canal through these holes using a T-handle.

**Figure 1:**
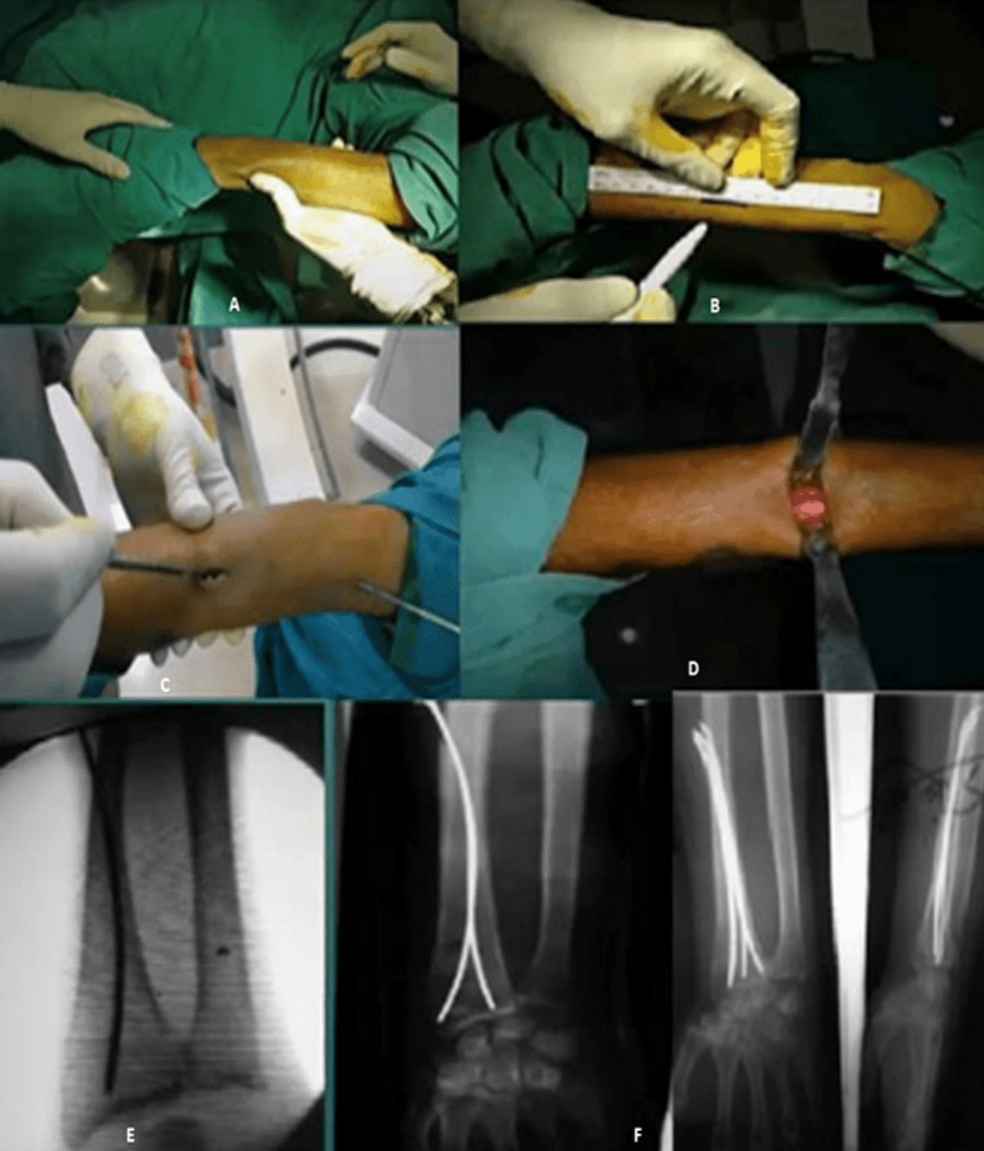
Operative procedure and postoperative X-ray. (A) Site of the incision. (B) Length of the incision. (C and D) Incision taken and soft-tissue dissection done to expose the bone. (E) C-arm picture of the bent K-wire insertion. (F) Postoperative X-ray image.

The pre-bent wires generated good curvature in the medullary canal during insertion. Initially, the wire tips were guided radially, and once reaching the fracture site, the distal fragment was manipulated while giving traction in the axis of the second and third digits, and counter-traction was given on the upper arm keeping the elbow flexed at 90° (Figure 1).

This was achieved in some cases by manual traction and in some by the mechanical method of traction. The reduction’s quality was then assessed using fluoroscopy in AP and lateral views by rotating the C-arm around the patient’s wrist while holding the patient’s hand steady. One intramedullary K-wire was rotated 180° and pushed to the ulnar side of the distal fragment, while the other wire was guided to the radial styloid. K-wires were advanced until they came into contact with the subchondral bone. The proximal ends of the wires were bent and buried in the subcutaneous tissue.

Postoperative protocol

Following surgery, a short-arm cast was applied for six weeks, allowing fingers and elbow motions to be free. After the removal of the cast, patients were advised to start physiotherapy exercises for wrist and forearm movements. K-wires were removed in the operating room under local anesthetic after an average of nine weeks (range: 8-12 weeks). At one, three, and six months of follow-up, all patients were clinically assessed for pain, functional status, range of motion, and grip strength. The afflicted wrist joint’s range of motion and grip strength were assessed and compared with those of the contralateral healthy wrist. Clinically, the wrist was examined using DASH scores, and the pain was measured using VAS grading.

The radiological examination included radial height and inclination measurements on AP and lateral radiographs before surgery, immediately after surgery, and at the six-month final follow-up. These measurements were utilized for the radiological assessment of patients based on radial height and inclination.

## Results

The mean age of patients was 45.6 years (18-80 years). The maximum number of cases were of the elderly age group with an age of >50 years (14/30 cases), followed by patients 31-40 years of age (7/30 cases).

The mechanism of injury in most cases was a high-velocity road traffic accident, followed by a fall from height. The female-to-male ratio was 3:2, with a female predominance. However, there was no statistical significance of sex in the present study.

In total, 14 patients suffered a fracture on their dominant hand, while 16 patients suffered an injury on their non-dominant hand. About 53.33% of patients presented within 24 hours to the hospital, while 10% of cases presented five days after injury and got delayed in treatment due to late presentation.

Overall 40% (12/30 cases) of the patients had a Frykman type I fracture, followed by 36.67% (10/30 cases) of patients with Frykman type II fracture. Further, 10% (3/30 cases) had type III and V injuries eachz, while only one case showed type VI injury. There were no cases of Frykman type IV injury.

The palmar flexion at the different follow-ups was analyzed. There was an average change from 43.5° at the first follow-up to 64° at the three-month follow-up, that is, the second follow-up, to 67° at the final follow-up. The dorsiflexion at the different follow-ups was also analyzed. There was an average change from 40.66° at the first follow-up to 58.67° at the three-month follow-up to 64.67° at the final follow-up Similarly, the improvement in supination was 35.33° at the first follow-up of one month to 60.16° at the three-month follow-up to the final 66.83° at six months. The improvement in pronation was 45.33° at the first follow-up of one month, 56.67° at the three-month follow-up, and 61.5° at six months. Adduction and abduction improved to 31.7° and 18.93°, respectively, postoperatively at the final follow-up of six months (Table 1).

**Table 1:** Distribution of participants according to the mean score (final outcome). DASH: Disabilities of Arm Shoulder and Hand; VAS: Visual Analog Scale

Parameter	Preoperative	One month	Three months	Six months	Chi-square	df	P-value
DASH score	82.44	59.76	24.72	6.62	205.0 8	16 5	0.018*
VAS score	7.93	3.86	2.30	1.3	16.80	9	0.019*
Radial height	7.66	11.20	10.56	10.30	11.1	12	0.52
Radial inclination	19.23	22.23	21.86	21.63	21.67	12	0.041*
Palmar flexion	-	43.5	64.00	67.00	10.44	12	0.57
Dorsiflexion	-	40.66	58.66	64.66	21.07	16	0.017*
Adduction	-	15.83	25.66	31.70	12.37	10	0.015*
Abduction	-	7	18.50	18.93	4.43	6	0.025*
Supination	-	35.33	60.16	66.83	9.72	12	0.63
Pronation	-	45.33	56.66	61.50	5.03	6	0.53

The range of movement improvement in the patients in the first and sixth month on applying the Mann-Whitney/Wilcoxon two-sample test revealed a statistically significant change in the outcome with less than 0.05 dorsiflexion, adduction, and abduction The average DASH score at the time of admission was 82.44, with a minimum of 75.8 and a maximum of 88.3. At the six-month follow-up, the average DASH score was 6.62 with a minimum of 5.2 and a maximum of 8.4. The average VAS score at the time of admission was 7.93. At the six-month follow-up, the average DASH score was 0.76 with a minimum of 0 and a maximum of 3.

Radiological assessment

The average radial inclination restored postoperatively was 21.63, with a maximum of 24 and a minimum of 20. The average radial length restored was 10.3 mm, with a maximum of 12 mm and a minimum height of 8 mm. The outcome from pre-intervention to the final follow-up, the radial height, and radial inclination were managed adequately at the time of operation and remained that way till the first follow-up after one month; however, they decreased in subsequent follow-ups. In the outcome from pre-intervention to final follow-up, the DASH score decreased from 82.44 to 6.62 (six-month follow-up). VAS score also decreased from 7.93 to 1.13, and both radial height and radial inclination increased. There was also an increment in palmar flexion, dorsiflexion, adduction, abduction, supination, and pronation from the first to the sixth month follow-up. DASH score, VAS score, radial inclination and dorsiflexion, adduction, and abduction showed significant p-values.

## Discussion

In this study, the mean age of our study patients was 46 years with maximum cases (14/30) aged more than 50 years, followed by patients aged 31-40 years (7/30 cases), with a male-to-female ratio of 2:3 showing a female dominance. There was no statistical significance in age and gender in this study, which points out that there was no bias in the selection of patients.

Overall, 40% (12/30 cases) of the patients had a Frykman type I, fracture, followed by 36.67% (10/30 cases) of patients with Frykman type II fracture, and 10% (3/30 cases) with types III and V injuries each, while only one case had type VI injury. There was no case of Frykman type IV injury. Similarly, in the study by Sato et al. [8], which included three men and 26 women with an average age of 58 years, fractures were graded as Frykman types I and II in 15 patients, types III and IV in 12 patients, and types V and VII in two patients.

Functional outcomes

In this study, palmar flexion at the different follow-ups was analyzed. There was an average change from 43.5° at the first follow-up to 64° at the three-month follow-up and to 72.83° at the final follow-up. Mean palmar flexion during the last follow was 64° with (chi-square: 1.12, p-value: 0.57). In dorsiflexion, there was an average change from 40.66° at the first follow-up to 58.67° at the three-month follow-up and to 64.66° at the final follow-up, with a mean of 58 degrees and a p-value of 0.017 which was highly significant. Similarly, the improvement in supination was 35.33° at the first follow-up of one month to 60.16° at the three-month follow-up to the final 66.83° at six months (p-value: 0.63). The improvement in pronation was 45.33° at the first follow-up at one month to 56.67° at the three-month follow-up to 61.5° at six months (chi-square: 9.72, p-value: 0.53) Adduction and abduction improved from 31.7° and 18.93°, respectively, postoperatively at the final follow-up of six months, with a mean of 31.7° and 18.93°, respectively (p-values: 0.015 and 0.025, respectively), which was highly significant.

Similarly, Clancey et al. [9] observed that 30 patients with displaced Colle fractures were treated with closed reduction along with percutaneous fixation with two K-wires. Their study showed that additional fixation improves anatomical and functional results after a displaced Colle fracture. Moreover, they recommended the use of percutaneous K-wire fixation if the articular surface of the radius is not comminuted into more than two fragments.

In our study, we assessed functional outcome by DASH score, unlike other studies where Mayo wrist scores by Azzopardi [10] and activities of daily living by Wong et al. [11] were used. In terms of DASH score, VAS score, radial inclination, dorsiflexion, adduction, and abduction, the improvement in functional outcome and range of movement in patients treated by K-wire was statistically significant.

A study by McQueenet et al. [12] showed that in elder individuals K-wires do not get enough hold in the osteopenic bone to maintain anatomical fracture reduction and improve function.

DASH Score

In our study, we assessed functional outcomes by DASH score. The average DASH score at the time of admission was 82.44 and decreased with every follow-up. The score was 6.62 at the final follow-up (chi-square: 205.08, p-value: 0.018) The improvement in functional outcomes and range of movement in the patients treated by K-wires was statistically significant.

A DASH score of 6.62 was achieved in six months in this study which was better than the study reported by Rampoldi et al. [13] in 2001 where the average DASH score was 30 points at three months and 14 points at six months.

VAS Score

In our study pain was assessed by VAS. Pain decreased by an average of seven points postoperatively. In the present study, the preoperative mean VAS score of the participants was 7.93 (SD: 0.86), and there was a decrease in the postoperative six-month follow-up mean which was 1.3 (SD: 0.85). It also shows the comparison of the participants based on preoperative and six-month VAS scores (chi-square: 16.80, p-value: 0.019), which was highly significant.

Radiological outcomes

In this study, the mean preoperative radial height was 7.66 mm, and after correction increased to 11.20 mm and finally fixed to 10.30 mm in the last follow-up (chi-square: of 11.1, p-value: 0.52). The radial inclination was corrected from 19.23 mm preoperatively to 22.23 mm on the first follow-up to 21.63 mm on the final postoperative follow-up, which showed an increment in the radial inclination with a p-value of 0.041 (significant) and a chi-square value of 21.67. These findings are consistent with the study done in 2010 by Wong et al. [11], which was a prospective randomized controlled clinical trial in a Chinese population of 60 patients to compare the functional and radiological effects of casting with percutaneous pinning in treating extra-articular distal radial fractures.

Sato et al. [8] reported a radial shortening value of 2.6 mm using antegrade intramedullary K-wire for fixation of the distal radial fracture, which is greater than the values in other studies. Mostafa et al. [14] observed a mean radial shortening of 0.96 mm which was lesser than that reported by Sato et al. in 2002. In our study, the mean radial height at the end of one month was 11.20 mm; however, on subsequent follow-ups, there was a decrease of 0.9 mm from one month to six months of follow-up.

This shows that antegrade intramedullary K-wires were effective in maintaining radial height as long as the wires were employed to support subchondral bone and unintentional articular surface penetration was avoided. However, on subsequent follow-ups, this technique failed to maintain the radial height. Additionally, raising the inclination angle of the entrance hole may improve the friction force between K-wires and the bone cortex, preventing the wires from backing out and collapsing at the fracture.

The K-wire group had statistically significant improved radiological outcomes in terms of dorsal angulation, radial inclination, and radial length. According to some research, closed reduction and retrograde percutaneous pinning may not produce adequate results. However, this study demonstrated that even with few comminuted fractures, excellent results can be obtained if enough reduction can be achieved using closed reduction.

Complications

Out of 30 patients, only three developed K-wire loosening during six weeks of follow-up. There were no signs of infections, skin irritation, and soft-tissue complications around the pins or Sudeck atrophy or CRPS. Sato et al. [8] reported a soft-tissue complication rate of 3% (one of 29 patients), which was lower than that of other studies.

Limitations

The main limitation of our study was that the sampling was limited to only one center and was small to conclude anything effectively, The functional outcome of the study was analyzed at 24 weeks, and larger comparative studies assessing the long-term results of the study are required. Due to the coronavirus disease 2019 pandemic, overall interactions and communications with patients were limited.

## Conclusions

Closed reduction and antegrade intramedullary K-wire fixation for reducible, unstable distal radius fractures was shown to achieve a good anatomical and functional outcome. Despite the small number of patients, the results showed that antegrade intramedullary K-wire fixation is an effective method for stabilizing distal radial fractures and preventing secondary displacement. This method was not appropriate for fractures with extensive intra-articular or metaphyseal comminution; therefore, patients must be carefully selected. For severely comminuted fractures, volar plating or external fixators are a better option.

The complications arising from the procedure were within acceptable limits. The morbidities arising from prolonged anesthesia were also avoided. This study emphasizes the necessity for a biomechanical study to determine how well intramedullary K-wire can endure axial, shearing, and bending forces. The financial impact on patients was less compared with other modes of operative treatment.
